# Fine-grained cell-type specific association studies with human bulk brain data using a large single-nucleus RNA sequencing based reference panel

**DOI:** 10.1038/s41598-023-39864-2

**Published:** 2023-08-10

**Authors:** Edwin J. C. G. van den Oord, Karolina A. Aberg

**Affiliations:** Center for Biomarker Research and Precision Medicine, Virginia Commonwealth University, McGuire Hall, Room 216A, 1112 East Clay Street, P. O. Box 980533, Richmond, VA 23298-0581 USA

**Keywords:** Transcriptomics, Computational biology and bioinformatics, Genetics, Neuroscience, Diseases

## Abstract

Brain disorders are leading causes of disability worldwide. Gene expression studies provide promising opportunities to better understand their etiology but it is critical that expression is studied on a cell-type level. Cell-type specific association studies can be performed with bulk expression data using statistical methods that capitalize on cell-type proportions estimated with the help of a reference panel. To create a fine-grained reference panel for the human prefrontal cortex, we performed an integrated analysis of the seven largest single nucleus RNA-seq studies. Our panel included 17 cell-types that were robustly detected across all studies, subregions of the prefrontal cortex, and sex and age groups. To estimate the cell-type proportions, we used an empirical Bayes estimator that substantially outperformed three estimators recommended previously after a comprehensive evaluation of methods to estimate cell-type proportions from brain transcriptome data. This is important as being able to precisely estimate the cell-type proportions may avoid unreliable results in downstream analyses particularly for the multiple cell-types that had low abundances. Transcriptome-wide association studies performed with permuted bulk expression data showed that it is possible to perform transcriptome-wide association studies for even the rarest cell-types without an increased risk of false positives.

## Introduction

Brain disorders such as mood disorders, dementias, stress related disorders, neurodevelopmental disorders, seizure disorders, and addictions are leading causes of disability worldwide^1^. Gene expression studies provide promising opportunities to better understand their etiology. The human brain comprises a diverse set of cell-types^2–4^. As these cells differ in their functions, gene expression will typically also vary across these cell-types. When studying bulk tissue, this cellular diversity may cause many genes that are differentially expressed in cases and controls to remain undetected^5^. That is, association signals will be “diluted” if they affect only one cell-type, may cancel out if they are of opposite signs across cell-types, and may be undetectable if they involve low-abundant cells.

Identifying the specific cell-types from which association signals originate is also critical for scientific progress and important from a translational perspective. First, it allows formulating refined hypotheses about disease etiology. For example, the involvement of microglia may point to disrupted immune response and neuroinflammation of the brain^6^, a loss of neuronal function may point to neurodegeneration^7^, and the involvement of the myelin-producing oligodendrocytes may suggest disrupted neuronal communication^8^. Second, knowledge about the cell-type is important to design proper in vitro or in vivo functional follow-up studies. Thus, as gene expression may only be altered in specific cells, such studies require the right choice of cultured cells or experimental tools (e.g., the use herpes simplex virus type 1 as a vector for locus-specific editing is of primary relevance for association findings in neurons^9,10^). Third, cell-type knowledge is key for developing novel and effective treatments. For example, drugs often work by interacting with receptors on the surface of cells. Receptor molecules have a specific three-dimensional structure, which allows only substances that fit precisely to attach to it. From a drug development perspective, designing drugs that interact specifically with receptors from particular cell-types is also highly desired since non-specific drugs can cause more side effects.

Capitalizing on deconvolution methods, cell-type specific associations can also be studied statistically using bulk RNA-seq data^5,11^. Deconvolution was introduced 20 years ago^11^ and has been experimentally validated using, for instance, predesigned mixtures^12^. Deconvolution is most effective when performed with a reference panel^13^. Reference panels comprise the expression profiles of the cell-types present in the target tissue that are typically generated from a small number of samples. The reference panel is used to estimate cell-type proportions in the bulk samples, which is in turn are used to perform cell-type specific association studies with bulk data. Reference panels can be created through expression profiling of sorted cells. However, while good nuclear protein markers exist for sorting nuclei into broad groups of neurons and glia, there is a lack of known, high fidelity, antigens and antibodies for further sorting subclasses of these brain cells. A better alternative is therefore to create the reference panel from single cell/nucleus RNA sequencing data^14^ that allows a fine grained analysis of brain cell-types. In comparison to whole cells, nuclei are more resistant to mechanical assaults and are less vulnerable to the tissue dissociation process. This makes single nucleus RNA sequencing (s_n_RNA-seq) the more suitable option for human post-mortem brain tissue^15^. With this approach intact nuclei are first isolated and partitioned so that the content of each nucleus can be labeled with a unique identifier. A labeled sequencing library is subsequently generated and sequenced for each individual nucleus.

A recent paper evaluated multiple reference panels and methods for estimating cell-type proportions for studies in brain^16^. However, several of the studied reference panels were small as they involved the first generation of s_n_RNA-seq studies, used specific donor (e.g., only male subjects) and age groups, focused on subregions of the prefrontal cortex, were not fine-grained or derived though a rigorous analysis of large s_n_RNA-seq studies. In this article we combined data from the seven largest published s_n_RNA-seq studies in the prefrontal cortex^17–23^, a brain region of key importance for higher level brain processes such as cognition, emotion, and memory. We derived the panel through an integrated analysis after processing all data in exactly the same way. A main advantage of this “mega-analysis” approach is that is reduces study specific technical artefacts. By focusing on cell-types robustly identified across the different studies, the panel will also have has more general applicability as it can be used across donor groups and brain regions. To estimate the cell-type proportions, needed to perform cell-type specific association studies with bulk data, we propose an estimator that can be used for a fine grained analysis of brain cell-types including multiple cell-types that are relatively rare. Finally, we study how to best use the proposed approach to optimize power and avoid false discoveries in empirical transcriptome-wide association studies with bulk data.

## Method

This section summarizes the methods. Details are given in the supplemental material (e.g., S1.1 refers to Sect.  1.1 in the supplemental material).

### s_n_RNA-seq data sets, quality control and data processing

We downloaded FASTQ files from seven published s_n_RNA-seq in post-mortem brain samples^17–23^. All brain regions involved the prefrontal cortex, predominantly from Brodmann areas BA6, BA8, BA9, BA10, and BA24. To avoid confounding the expression values in the panel by disease processes or disease specific cell states, only the unaffected “controls” from these studies were used.

All seven studies partitioned nuclei using the Chromium Controller (10X Genomics) and sequenced the libraries on Illumina platforms. We used the cellranger^24^ software for aligning the reads to GRCh38 and creating a matrix of unique molecular identified (UMI) counts (i.e., the number of unique molecules for each gene detected in each nucleus). s_n_RNA-seq data primarily yields reads derived from mature spliced RNA (mRNA), which maps to exonic regions but may also capture unspliced pre-mRNA transcripts that can generate intronic reads^25–27^. As nuclei contain a relatively large fraction of pre-mRNA molecules and such molecules are particularly abundant in brain tissue^28^, to obtain a comprehensive picture of gene expression we counted intronic reads as well^29^.

We performed quality control (QC) on samples and nuclei using exactly the same criteria across all studies. Specifically, we eliminated samples with very high levels of debris (Figure S1). In addition, we removed nuclei with very low (indicating low-quality nuclei or empty droplets) or high (indicating “multiplets” that capture expression levels of multiple nuclei) gene and UMI counts (Figures S2 and S4). Finally, nuclei with a high percentage of reads mapping to mitochondrial genes (possible indicating artifacts stemming from sample preparation) were eliminated.

For each study separately, the QC’ed count data was log-normalized to obtain more normal distributions and reduce effects of possible outliers. Furthermore, to avoid that highly expressed genes dominate the cluster analyses, genes were given equal weight by scaling the log-normalized count data to have a mean of zero and a standard deviation of one.

### Clustering

To identify cell-types, we performed a cluster analysis in Seurat^30^ (S.1.1). We "anchored" the different datasets in a shared cluster space to facilitate their integration^31^. The cluster analysis was limited to the 2,000 genes that exhibited the highest nucleus-to-nucleus variation (i.e., highly expressed in some nuclei and lowly expressed in others)^32^. There are potentially a large number of donor-level covariates (e.g., medication use, cause of death, cDNA yield, post-mortem interval) that may obscure the separation of clusters. However, as many nuclei are assayed from the same donor, we can remove the effects of donor-level confounders by controlling for the factor “donor”. Technically this was achieved by regressing out “indicator” variables for the donors (i.e., for each donor these is one variables that has a value of 1 for that donor and is zero for all other donors). Thus, the data are analyzed as deviations from the donor specific means so that any variable that contributes to differences between donors will no longer affect the measurements. To control for nuclei-level confounding, we also regressed out the QC indices listed above.

### Deconvolution

Deconvolution involves three steps. First, a reference panel^33,34^ is created (S1.2). To select genes for the panel, we used MAST^35^ that performs significance tests to identify the genes that best discriminate between the cell-types. The expression values from the s_n_RNA-seq data were scaled to have a mean of zero and variance of one for each study, and then an average expression value was computed across all studies. This was done to avoid that the panel was dominated by specific studies (e.g., large studies) and ensure that the derived cell-types were robustly identified across all studies.

Second, the reference panel in combination with the bulk RNA-seq data is used to estimate cell-type proportions in each bulk sample. To estimate the cell-type proportions, we use the standard linear model^36^ but estimated by empirical Bayes^37^ (EB) to enable precise estimation of potentially multiple low abundant cell-types (for estimation details see S1.3.1 and R code is provided at https://github.com/ejvandenoord/Empirical-Bayes-estimation-of-cell-type-proportions). The mean and the standard deviation of estimates produced by fitting the same model subject to a non-negativity constraint for the regression coefficients (i.e., the cell-type proportions) was used as the prior distribution. A recent paper^16^ performed a comprehensive evaluation of methods to estimate cell-type proportions from brain transcriptome data. Based on their performance, the authors recommended CIBERSORT^38^, dtangle^39^ or MuSiC^40^. To evaluate our estimator, we compared it with these three methods. For CIBERSORT we the latest version, called CIBERSORTx^41^, as used for the web version of the software.

Third, the estimated cell-type proportions are used to perform cell-type specific association studies with bulk data, This is done by fitting, for each transcript, the model as described elsewhere^12^ (see also S1.3.2). These association analyses were performed using the Bioconductor package RaMWAS^42^.

### Demonstration bulk RNA-seq dataset

Bulk RNA-seq data was generated using tissue from BA10 of from 291 control individuals and 304 individuals that were diagnosed with a psychiatric disorder (S1.4). All experimental protocols were approved by the local IRB at Virginia Commonwealth University and informed consent was obtained from all subjects and/or their legal guardian(s). All methods were performed in accordance with the relevant guidelines and regulations by including a statement in the methods section. The RNA-seq data was generated using the TruSeq Stranded Total RNA library kit. The sequenced reads were aligned with HISAT2 (v.2.1.0) and transcriptome assembly was performed with StringTie^43^. All analyses (i.e., cell-type proportion estimation and deconvolution analyses) regressed out the covariates: sex and age, indicator variables to account for possible brain banks effects, and assay-related covariates such as total number of reads and the percentage of reads aligned. Furthermore, to account for remaining unmeasured sources of variation, six principal components (as suggested by the scree plot) that were used as covariates after regressing out the measured covariates from the bulk RNA-seq data.

## Results

### Sample description and QC

The seven studies included s_n_RNA-seq data from 94 unaffected “control” subjects. The mean age was 61.6 years (SD = 28.6 years) with the 5th/95th percentiles of 12.7/90.0 years indicating a very broad range. The subjects comprised 37% females. The post-mortem interval was 19.6 h (SD = 15; 5th/95th percentile of 2.5/49.4 h).

Table S1 lists assay related statistics. In summary, we observed an average of 65,118 reads per nucleus. Of these reads, 93.6% mapped to the genome with 78.8% of reads having nucleus-associated barcodes. Using the same criteria for all seven studies, we quality controlled samples and nuclei (S2.1, Figures S1–S5). Two studies had many more nuclei per donor (34,342 and 22,831 nuclei) than the other five studies (mean 5154 nuclei). To avoid that the clustering was mainly driven by these two studies, we down-sampled their nuclei to 8,562 and 8,567 to obtain an average of 5,547 nuclei (range 1426–10,039 nuclei) across all seven studies. After QC and down-sampling, 353,146 nuclei from 92 donors remained.

### Clustering and cell-type labeling

**Figure 1:**
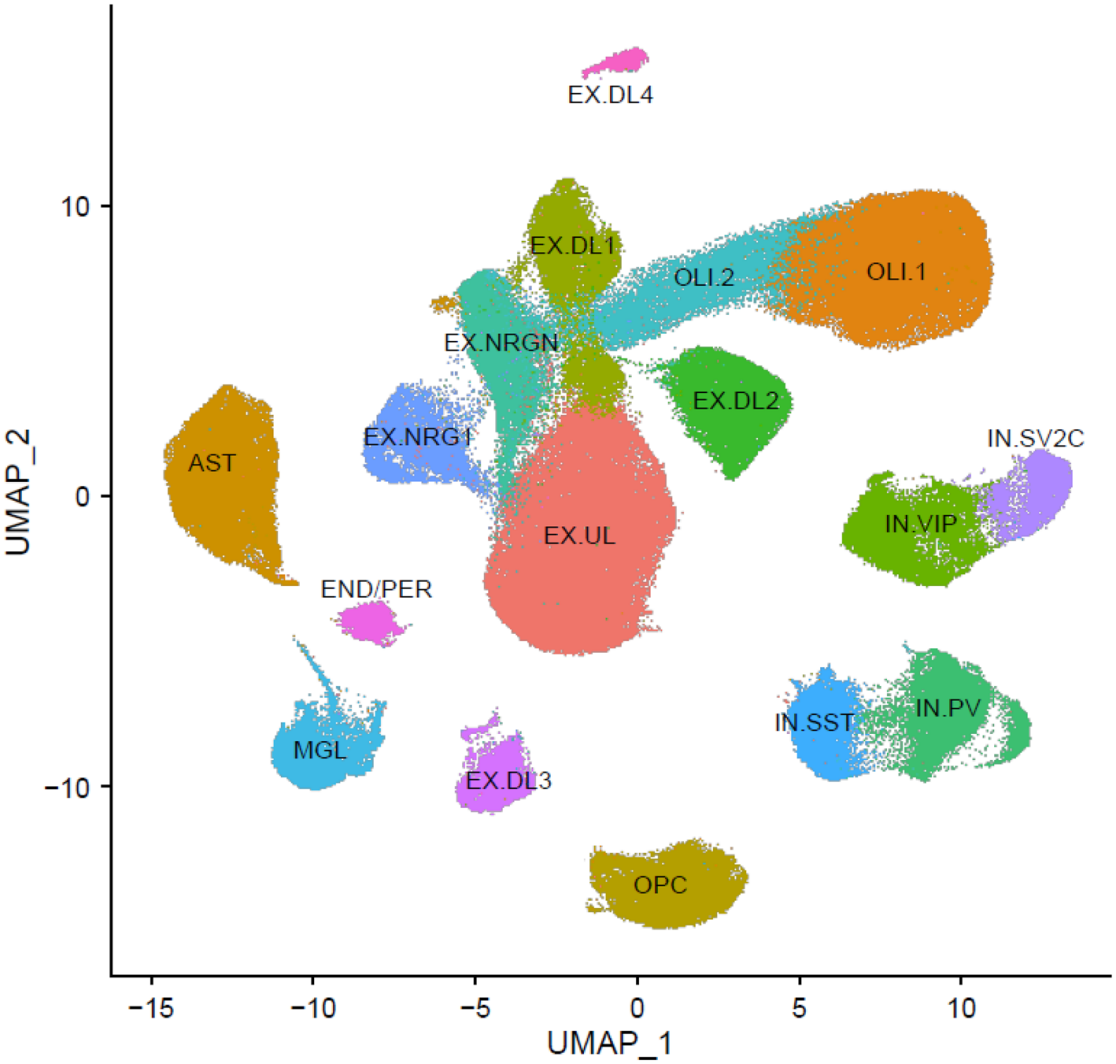
A Uniform Manifold Approximation and Projection (UMAP) plot depicting the nuclei of the 17 clusters. The cluster labels are described in the main text.

Clustering identified 20 groups of nuclei, 17 of which were observed in all seven studies. The three clusters that were not consistently observed were removed from further analyses. Figure 1 visualizes the cell-type clusters. To plot the clusters, which differ on many dimensions, in a two-dimensional space we used Uniform Manifold Approximation and Projection (UMAP).

Figure S6 provides a dotplot for the markers used to label the cell-types and Figure S7a–e shows heatmaps of the overlap between the cell-type labels assigned in this study and the label assigned in the five of the seven original studies that provided nuclei labels. These results are further summarized in Table S2 provides for each of our cell-type clusters a list of the most highly expressed markers as well as the most frequently assigned original cell-type label in the five studies that provided labeled nuclei.

Of the 17 clusters, 14 could readily be labeled using standard markers. Although it should be noted that only two studies attempted labeling subtypes of broad groups of nuclei (e.g., excitatory neurons), the nuclei of the 14 clusters were consistently labeled by the five studies that provided the original cell-type labels. These 14 clusters included one of the two clusters of oligodendrocytes (OLI.1)^44^, oligodendrocyte precursor cells (OPC)^44^, astrocytes(AST)^45^ and microglia (MGL)^46^. Four clusters of interneurons (IN) were identified that could further be labeled based on the expression of somatostatin (IN.SST), parvalbumin (IN.PV), vasoactive intestinal peptide (IN.VIP), and synaptic vesicle glycoprotein 2C (IN.SV2C)^47^. Finally, seven groups of excitatory neurons were identified. These neurons were further subdivided into one cluster of upper-layer (EX.UL) neurons and four clusters of deep-layer (EX.UL1-EX.UL4) neurons all expressing FOXP2 and subsets of other standard layer-specific markers. Furthermore, we observed neurons expressing neurogranin (EX.NRGN).

Three clusters could not unequivocally be labeled with standard markers and were also inconsistently labeled across the five studies that provided labels for individual nuclei. First, we observed a cluster expressing standard markers for both endothelial cells^48^ and pericytes^37^. In the original studies these nuclei were labeled as endothelial cells^48^, pericytes ^37^, or as a combined cluster of endothelial cells and pericytes. As these nuclei most likely included both endothelial cells and pericytes that have very similar expression profiles relative to the other clusters in Fig. 1, were labeled this cluster END/PER.

Second, albeit at relative modest levels compared to OLI.1, the second cluster of oligodendrocytes (OLI.2) expressed standard oligodendrocytes markers MBP, PLP1, and MOBP. In addition, we observed the expression of NRGN, CAMK2A, and CAMK2B that share a motif with MBP potentially allowing it to be packaged together for cytoplasmic transport to dendrites^49^. Three studies labeled these nuclei as oligodendrocytes and the other two studies as neurons. Neurons can use the same packaging mechanism for cytoplasmic transport of the RNAs to dendrites and this potentially explains the confusion about the identity of this second group of oligodendrocytes.

Third, a cluster of EX neurons expressed only few of the markers expressed by the other EX clusters and was inconsistently labeled with respect to cortical layer in the two studies that labeled EX subtypes. This EX cluster expressed NRG1 at very high levels (EX. NRG1). NRG1 is expressed in multiple cell-types and best known as a gene affecting a range of psychiatric and neurological disorders such as Alzheimer, autism and schizophrenia^50,51^. To learn more about the identity of this cluster, we selected the ten most highly expressed genes from the reference panel. Six of the ten genes were previously reported to be associated with a range of psychiatric and neurological disorders. In addition to NRG1^50,51^, this included ZNF804B^52^, CDH12^53^, CLSTN2^54,55^, RIT2^56^, and MCTP1^57^. This pattern is somewhat reminiscent of so-called Von Economo neurons (VENs) that are known to be altered in diseases such as Alzheimer, autism, and schizophrenia^58–60^. VENs are found in humans and great apes (but not other primates), cetaceans, and elephants, and may have evolved for the rapid transmission of crucial social information in very large brains^61^. In humans, VENs are abundant in the anterior cingulate and frontoinsular cortices but are also present in the prefrontal cortex^62^. A recent study involving 879 nuclei from frontoinsula layer 5 identified several VEN markers, but these markers were not highly expressed in our cluster.

### Cell-type proportion estimation

Table S3 gives the MAST^35^ test results identifying 1,652 genes for the reference panel (Table S4). The EB estimation procedure was first evaluated using artificial bulk data. We generated artificial bulk data using the cell-type specific expression values from the panel in combination with cell-type proportions that were randomly drawn from a generalized beta distribution assuming the mean, standard deviation minimum, and maximum of the cell-type proportions observed in our demonstration bulk RNA-seq dataset.

CIBERSORTx Table 1 shows a comparison of the EB methods with methods previously recommended after a comprehensive evaluation of methods to estimate cell-type proportions from brain transcriptome data^16^. The MuSiC package is specifically designed for s_n_RNA-seq data. We had to down-sample the number of nuclei to avoid excessive run times. We could also not replicate our previous observation that MuSiC produces superior estimates^63^. Instead, results were so poor that they likely indicated convergence problems so that this estimator was not further considered. Table 1 shows that all estimators were unbiased. However, the EB estimator was substantially more precise. Thus, compared to EB the RMSEs for CIBERSORTx and dtangle were five (0.025 vs. 0.005) and 6.8 (0.034 vs. 0.005) times larger. This increased precision translated to systematically higher correlations (0.936 vs. 0.846 and 0.784 for CIBERSORTx and dtangle) between the true and estimated cell-type proportions. These correlations remained satisfactory even for rare cell-types. Although cell-type proportions close to zero can be estimated at zero by chance, a large number of zeroes may indicate problems with estimating low abundances precisely. CIBERSORTx produced a relatively large number of zeroes particularly for low abundant cell-types. In contrast, dtangle did not produce any zeroes but as the other indices indicated that this estimator had the lowest precision this may be interpreted as meaning that it estimates low abundances precisely.

**Table 1:** Evaluate the empirical Bayes estimation procedure.

	CIBERSORTxx				dtangle				Empirical Bayes			
	Bias	RMSE	Zero	Cor	Bias	RMSE	Zero	Cor	Bias	RMSE	Zero	Cor
EX.UL	0.010	0.040	8	0.911	0.077	0.082	0	0.920	− 0.002	0.006	0	0.985
OLI.1	0.001	0.067	167	0.924	0.056	0.082	0	0.815	0.003	0.014	12	0.979
AST	0.001	0.038	105	0.952	0.045	0.057	0	0.989	0.000	0.005	1	0.993
OPC	0.008	0.017	26	0.873	0.024	0.026	0	0.855	− 0.001	0.003	0	0.968
EX.DL1	0.003	0.019	33	0.829	− 0.015	0.018	0	0.652	− 0.001	0.004	0	0.933
IN.VIP	0.003	0.019	53	0.876	0.000	0.012	0	0.824	− 0.001	0.004	0	0.964
EX.DL2	− 0.002	0.023	57	0.889	− 0.009	0.019	0	0.837	− 0.001	0.005	0	0.963
IN.PV	0.001	0.019	53	0.908	− 0.017	0.022	0	0.845	− 0.001	0.004	0	0.969
EX.NRGN	− 0.022	0.057	320	0.932	− 0.024	0.055	0	0.930	0.002	0.006	90	0.995
OLI.2	− 0.016	0.049	274	0.912	− 0.010	0.043	0	0.640	0.001	0.011	57	0.972
MGL	0.004	0.013	119	0.926	0.013	0.017	0	0.931	0.000	0.002	0	0.983
IN.SST	0.003	0.014	61	0.790	− 0.020	0.021	0	0.632	0.000	0.003	0	0.897
EX.NRG1	0.004	0.014	103	0.583	− 0.054	0.054	0	0.650	0.000	0.004	0	0.784
IN.SV2C	0.002	0.009	60	0.718	− 0.006	0.007	0	0.635	0.000	0.002	0	0.860
EX.DL3	0.002	0.011	193	0.674	− 0.047	0.048	0	0.541	0.000	0.003	0	0.811
END/PER	− 0.001	0.009	265	0.896	− 0.001	0.008	0	0.911	0.000	0.002	11	0.976
EX.DL4	− 0.001	0.007	209	0.783	− 0.010	0.011	0	0.715	0.000	0.002	18	0.883
Average	0.000	0.025	124	0.846	0.000	0.034	0	0.784	0.000	0.005	11	0.936

RMSE is the root of the means squared error, Zero is the number of cell-type proportions estimated at zero, and Cor. is the correlation between true and estimated cell-type proportions.

We also studied the performance under less ideal circumstances. That is, to simulate a mismatch between panel and bulk data, we replaced 50% of all bulk gene expression data with a random value and also increased the error in the bulk data by a factor 10. Table 2 shows that the pattern of results mimicked those from Table 1. Although the RMSE and correlation decreased the EB seemed robust producing estimates that could still be used in research.

**Table 2:** Relative robustness of the empirical Bayes estimation procedure.

	Bias	RMSE	Zero	Cor
Baseline				
CIBERSORTxx	− 3.4E−18	0.025	124	0.846
dtangle	7.1E−19	0.034	0	0.784
Empirical Bayes	1.3E−18	0.005	11	0.936
50% of bulk values are random				
CIBERSORTxx	− 1.4E−18	0.017	84	0.817
dtangle	1.1E−18	0.034	0	0.754
Empirical Bayes	2.4E−18	0.021	65	0.894
Multiply error in bulk data by 10				
CIBERSORTxx	2.1E−18	0.035	176	0.649
dtangle	− 1.0E−19	0.034	0	0.523
Empirical Bayes	2.0E−19	0.010	11	0.761

RMSE is the root of the means squared error, Zero is the number of cell-type proportions estimated at zero, and Cor. Is the correlation between true and estimated cell-type proportions.

### Cell-type specific association studies

**Figure 2:**
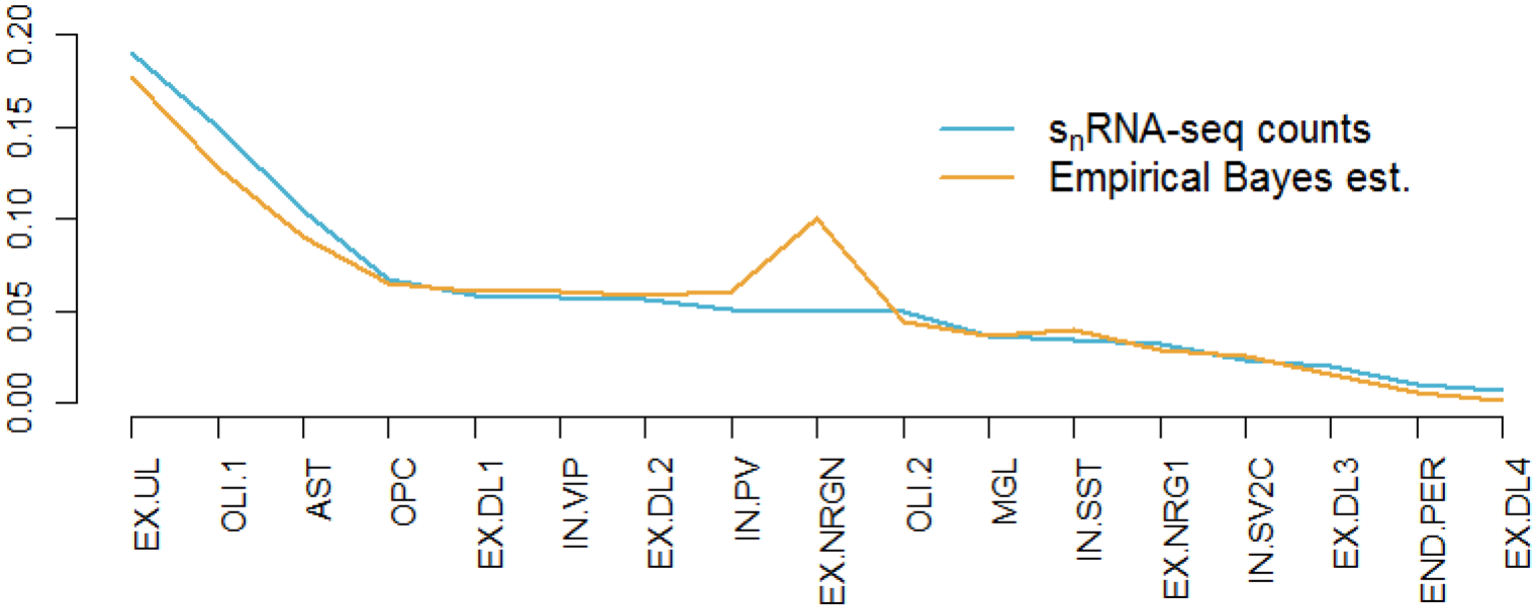
Mean cell-type frequencies observed in the s_n_RNA-seq data and estimated in bulk samples.

Figure 2 shows that the mean of the cell-type proportion estimates in our demonstration bulk RNA-seq dataset was highly correlated with the mean s_n_RNA-seq counts (*r* = 0.951). Only EX.NRGN showed a notable difference. Given that our simulation study yielded unbiased estimates, this most likely reflects true biological variation between the two sample sets. Similar to what we observed in the simulation study, even for rare cell-types few estimates were estimated to be zero (average 3.2%).

**Figure 3:**
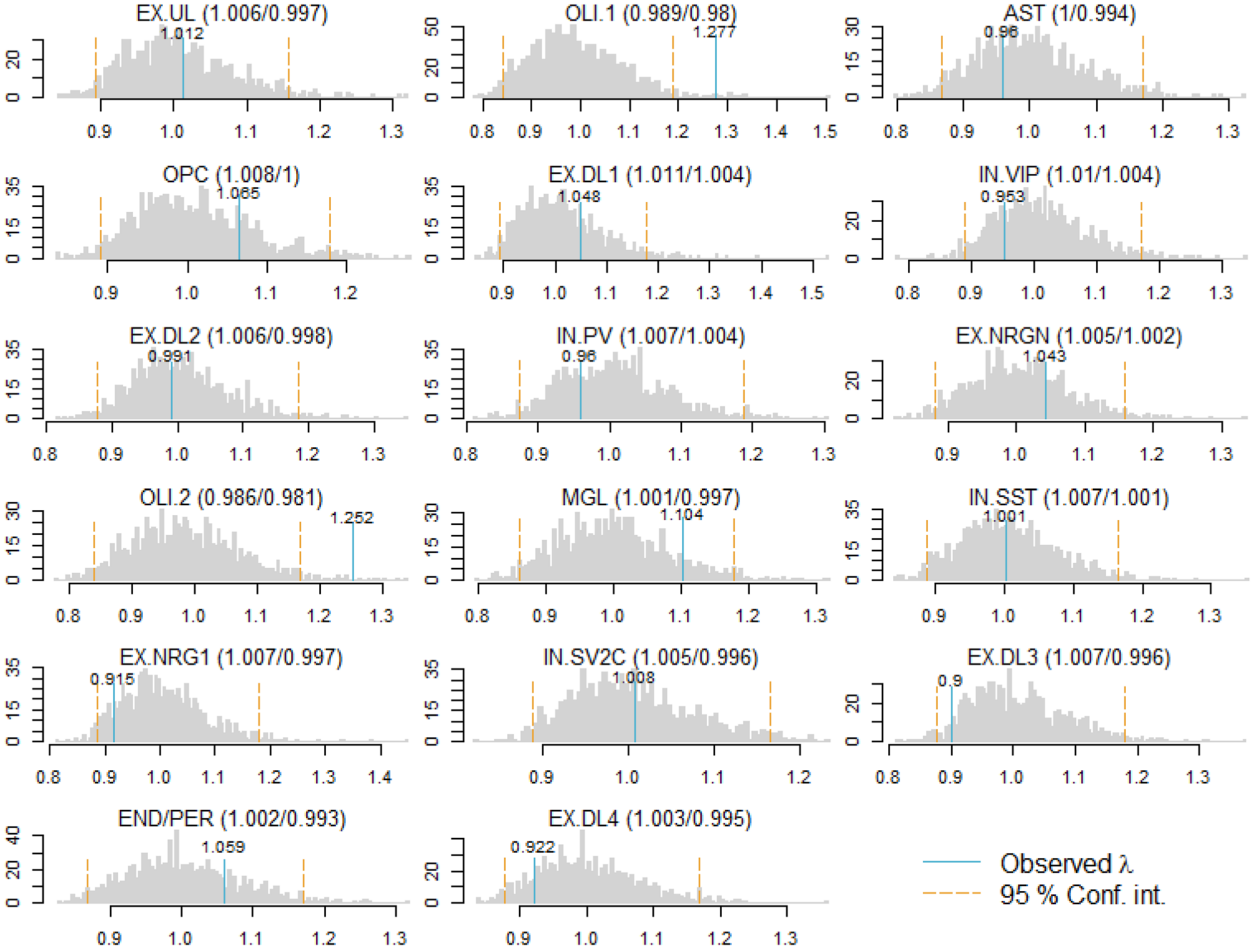
Histograms of observed and simulated lambdas.

To study whether the distribution of the tests statistics under the null hypothesis followed the assumed theoretical distribution, 1,000 transcriptome-wide association studies (TWASs) where performed after randomly permuting case–control labels. Results showed that for each cell-type lambda (ratio of the median of the observed distribution of the test statistic to the expected median) was close to one (Fig. 3, overall median/mean 1.01/1.03 with range 0.90–1.28). This implied the absence of test statistic inflation and that under the null distribution accurate *P* values are obtained. This was true for even the rarest cell-types suggesting that it is possible to perform TWASs on rare cell-types without an increased risk of false positives.

Overall 11 genes were transcriptome-wide significant when controlling the FDR controlled at the 0.1 level and 105 findings reached “suggestive” significance when controlling the FDR controlled at the 0.1 level FDR controlled at the 0.25 level (i.e., meaning that 10 and 25%, respectively, of the findings are expected to be false).

We studied whether power could be improved by grouping similar cell-types. For this purpose, we performed a principal components analysis (PCA) followed by a varimax rotation on the gene expression values of the panel. Cell-types with loadings > 0.5 on the same principal component were combined. The PCA suggested that six groups of two cell-types (Table S5) could potentially be combined leaving 11 cell-types. These PCA results corresponded very well with the UMAP plot (Fig. 1) showing proximity of these same cell-types. After grouping 68 genes reached transcriptome-wide significance when controlling the FDR controlled at the 0.1 level and 106 findings reached “suggestive” significance when controlling the FDR controlled at the 0.1 level FDR controlled at the 0.25 level. This the signal improved most likely because in this demonstration dataset the combined cell-types showed similar association signal.

## Discussion

We propose a new brain reference panel that allows the detection of differentially expressed genes in human bulk brain data on a fine-grained cell-type specific level. We created the panel through an integrated (mega) analysis of the data from the seven largest s_n_RNA-seq studies in brain after processing all data in exactly the same way. Our panel included 17 cell-types that were robustly detected across all studies, subregions of the prefrontal cortex, and sex and age groups.

Our goal was not to make a complete inventory of all cell-types in the PFC but to create a reference panel that can subsequently be used for cell-type specific association studies. Thus, cell-types that were not observed in all studies were omitted and we used settings in the cluster analyses that would avoid a very large number of cell-type clusters. We believe that the proposed panel strikes a good balance between being fine-grained but not to the extent that all the cell-type proportions can no longer be estimated precisely.

To estimate the cell-type proportions, we proposed an empirical Bayes estimator that yielded highly accurate and unbiased estimates even for the low abundant cell-types. Our estimator substantially outperformed the three estimators recommended previously after a comprehensive evaluation of methods to estimate cell-type proportions from brain transcriptome data^16^. Our panel contains a substantial number of cell-types, several of which had low abundances. A precise estimator is critical as to avoid that downstream analyses with low abundant cell-types produce unreliable results. Furthermore, our estimator has the desirable property that it uses a panel comprising mean expression levels rather than the nuclei level s_n_RNA-seq data. This prevents working with very large data files and the need to apply for access to obtain all the nuclei level data from repositories.

Transcriptome-wide association studies performed with permuted bulk RNA-seq data showed that it is possible to perform TWASs for even the rarest cell-types without an increased risk of false positives. For example, even cell-types with frequencies as low as 1% yielded transcriptome-wide significant results in the absence of test statistic inflation. Furthermore, analyses showed that more significant findings were obtained by grouping similar cell-types. How, this finding may be specific for our demonstration dataset and it is very well possible that in other data sets fewer significant findings are obtained when grouped cell-type so not show associations with the same genes.

The proposed approach requires bulk gene expression data to estimate the cell-type proportions. However, once the cell-type proportions are estimated, we can study cell-type specific associations for any other bulk data generated for the same brain samples (e.g., microRNAs, methylation data, open chromatin data). Although s_n_RNA-seq studies, and consequently our panel, involve gene expression, we can therefore also study individual transcripts. This is important as only specific transcripts of the gene may be differentially expressed. In these scenarios the study of expression at the gene level will dilute association signals and result in a loss of potentially critical biological information.

Whereas s_n_RNA-seq assays nuclear RNAs with a poly A-tail (mainly mRNA), our bulk RNA-seq data assayed total RNA from the entire cells which may contain transcripts not present in the nucleus^64,65^. However, the means of the cell-type proportion estimates in our bulk RNA-seq dataset were highly correlated with the cell-type proportion counts observed in the s_n_RNA-seq data. This suggested that possible differences in expression levels between the panel genes in the nucleus versus the entire cell did not distort the cell-type proportion estimates.

Even with the advent of s_n_RNA-seq, deconvolution methods are likely to remain pertinent for cell-type specific association studies with brain tissue. This is because the vast majority of existing gene expression data sets involves bulk samples. Deconvolution allows the (re-)use of this “legacy” data to study cell-type specific effects. Deconvolution methods can also potentially be useful to validate findings from s_n_RNA-seq studies. Such validation with a different technology can eliminate possible false discoveries due to technology specific artefacts and therefore allows for more rigorous conclusions.

In summary, brain disorders are leading causes of disability world-wide. We proposed a new reference panel and precise estimator for the cell-type proportions that allows the use of bulk brain data to study brain disorders on a fine-grained cell-type specific level. The use of this approach may prevent that many cell-type specific disease associations remain undetected in studies with bulk data. Furthermore, identifying the specific cell-types from which association signals originate is key to formulating refined hypotheses about the etiology of brain disorders, designing proper follow-up experiments and, eventually, developing novel clinical interventions. The reference panel and easy-to-use accompanying analysis tools are publicly available.

### Supplementary Information

Supplementary Information 1.

Supplementary Information 2.

Supplementary Information 3.

Supplementary Information 4.

Supplementary Information 5.

## Data Availability

The datasets during the current study are available in the GEO (accession numbers GSE157827, GSE138852, GSE174367,GSE144136, GSE144136, GSE140231) and Synapse (accession number syn18642926). The panel and R scripts used for empirical Bayes estimation and its evaluation though simulations are available from GitHub: https://github.com/ejvandenoord/Empirical-Bayes-estimation-of-cell-type-proportions. RaMWAS is freely available from Bioconductor (https://bioconductor.org/packages/release/bioc/html/ramwas.html) and a script to perform cell-type specific association studies with RaMWAS is also provided on GitHub https://github.com/ejvandenoord/celltype_MWAS.
