# Supplementary material for: Fine-grained cell-type specific association studies with human bulk brain data using a large single-nucleus RNA sequencing based reference panel
Source: Sci Rep. 2023 Aug 10;13:13004. doi: 10.1038/s41598-023-39864-2 (PMC10415334; doi:10.1038/s41598-023-39864-2)
Supplement: Supplementary file 1 — Supplementary Information 1. [file 41598_2023_39864_MOESM1_ESM.docx]

SUPPLEMENTAL MATERIAL FOR THE PAPER “Fine-grained cell-type specific association studies with human bulk brain data using a large single-nucleus RNA sequencing based reference panel” by van den Oord and Aberg

Contents

[S1 METHODS 2](#_Toc131511111)

[S1.1 Clustering 2](#_Toc131511112)

[S1.2 Creating the reference panel 2](#_Toc131511113)

[S1.3 Cell-type specific association studies with bulk data 2](#_Toc131511114)

[S1.3.1 Estimating cell-type proportions 2](#_Toc131511115)

[S1.3.2 Cell-type specific association studies with bulk data 3](#_Toc131511116)

[S1.4 Demonstration bulk RNA-seq dataset 4](#_Toc131511117)

[S2 RESULTS 4](#_Toc131511118)

[S2.1 Alignment and quality control (QC) 4](#_Toc131511119)

[Table S1. Sequencing statistics (separate file) 4](#_Toc131511120)

[Figure S1 Examples of barcode rank plots 5](#_Toc131511121)

[Figure S2. Distribution and QC threshold for number of genes per nucleus 6](#_Toc131511122)

[Figure S3. Violin plot for number of genes per nucleus per cell-type 7](#_Toc131511123)

[Figure S4. Distribution and QC threshold for number of UMI counts per nucleus 8](#_Toc131511124)

[Figure S5. Violin plot for number of UMI counts per nucleus per cell-type 8](#_Toc131511125)

[S2.2 Cell-type identification and labeling 9](#_Toc131511126)

[Figure S6. Dotplot of gene expression markers used to annotate the clusters 10](#_Toc131511127)

[Figure S7a-e. Heatmaps of the percent of overlap between cluster labels 11](#_Toc131511128)

[Table S2. Cell-type labeling (separate file) 13](#_Toc131511129)

[Table S3. MAST identified cell-type panel markers (separate file) 14](#_Toc131511130)

[Table S4. Cerebral cortex reference panel (separate file) 14](#_Toc131511131)

[Table S5. Grouping cell-type by principal components analysis 14](#_Toc131511132)

[REFERENCES 14](#_Toc131511133)

# S1 METHODS

### S1.1 Clustering

We used Seurat(1) to identify clusters of nuclei with similar expression profiles. We used integrative analyses(2) that first identifies matching nuclei between the groups called ‘anchors’ followed by clustering all nuclei in the shared space defined by the anchors. To improve results from cluster analyses(3), we limited analyses to the 2,000 genes that exhibited the highest nucleus-to-nucleus variation (i.e., highly expressed in some nuclei and lowly expressed in others). Donor-level variation (e.g., due to demographic variables) and confounders (e.g., cDNA yield, percentage of reads aligned) may obscure the separation of clusters. However, as many nuclei are assayed from the same donor, we can remove the effects of donor-level confounders by controlling for the factor “donor”. Technically this was achieved by regressing out “indicator” variables for the donors (i.e., for each donor these is one variables that has a value of 1 for that donor and is zero for all other donors). Thus, the data are analyzed as deviations from the donor specific means so that any variable that contributes to differences between donors will no longer affect the measurements. In addition, we regressed out the QC measures discussed below (S2.2, i.e., log 10 number of genes per nucleus, log 10 UMI counts per nucleus, and % of reads mapping to ribosomal genes). Next, we constructed a K-nearest neighbor graph based on the Euclidean distance of the space defined by the 15 principal components (PCs) that explained most of the variation in the data, and further refined the edge weights between any two nuclei based on the shared overlap in their local neighborhoods. The resolution parameter was set to 0.2 as that gave a robust set of well-delineated clusters. Uniform Manifold Approximation and Projection (UMAP) was used to visualize the cell-type clusters in two-dimensional space. Compared to linear techniques, UMAP may provide a clearer picture of the clusters as nuclei that are close to one another in the original higher-dimensional space are more likely to remain be close to one another in the low two-dimensional space.

### S1.2 Creating the reference panel

MAST(4) was used to select only the most informative genes for the panel that best discriminate between the identified clusters. We used MAST to select genes from either all genes or only the 2,000 highly variable genes that were used to create the clusters. Results suggested that starting with only the 2,000 highly variable genes gave the best results and that approach was used to create the panel. Prior to creating the panel, we eliminated nuclei that were outliers with respect for the cell-type they were assigned to. For this purpose, we calculated for each nucleus an outlier score that was the mean of the absolute differences between all the feature means for that nucleus and the corresponding feature means across all nuclei of that cell-type. Next, we calculated the median absolute deviation (MAD) of these outlier scores and identified outliers as nuclei with absolute( outlier score - median( outlier score)) / MAD( outlier score) > 3. We use the MAD rather than standard deviations to define outlier as the MAD is the more robust measure(5).

### S1.3 Cell-type specific association studies with bulk data

Cell-type specific association studies with bulk data using deconvolution methods involve two steps. We first estimate the cell type proportions for each sample and then use these estimates to test, for each cell type separately, the null hypothesis that the mean expression of a given gene is equal for cases and controls.

### S1.3.1 Estimating cell-type proportions

Gene expression in bulk tissue is a weighted sum of the average expression levels of each cell type with weights being equal to the proportion of cells of each type. Thus, if we know the expression levels for each of the $c=1..n_{c}$ cell types we can calculate bulk expression levels as follows:

$$\bar{Y}_{i}^{bulk}=\sum_{c=1}^{n_{c}} p_{i}^{c}T_{i}^{c}$$

Where the *m*×1 vector $\bar{Y}_{i}^{bulk}$ contains the bulk expression levels of the *m* genes of donor *i*, *c*=1..*n*_c_ indicates the cell types, is the proportion of cells of type *c* for donor *i*, and $T_{i}^{c}$ is a *m*×1 vector of cell type *c* specific expression levels of the *m* genes of donor *i*. The cell type proportions $p_{i}^{c}$can be estimated using a “reference” panel using the following regression model originally proposed by Houseman(6, 7):

:

$Y_{i}^{bulk}=\sum_{c=1}^{n_{c}} p_{i}^{c}R^{c}+E_{i}$ (1)

Where the *m*×1 vector $Y_{i}^{bulk}$ represents the *m* expression measurements for donor *i* in bulk tissue, are the regression coefficient for cells of type *c* for donor *i*, the *m*×1 vector *R*^c^ contains for each genes the mean expression on the reference panel for cell type *c*, and *E_i_* is a *m*×1 vector with residuals. Thus, the expression levels assayed in bulk tissue for donor *i* is again a weighted sum of the average expression levels of the cell type specific methylomes but we now replaced replaces the unknown cell type specific expression values $T_{i}^{c}$ with the corresponding values *R*^c^ that are known. The weights are the estimated coefficients of the regression model and correspond again to the proportion of cells of each type. The reference panel will not perfectly match the true cell type expression profiles for donor *I* and the model accounts for that through the estimated residuals.

To estimate the cell-type proportions, we use the model in Equation1 but we impose the constraint that all regression coefficients (i.e., cell-type proportions) are larger than zero. Furthermore, rather than using ordinary least squares, the regression coefficients are estimated by empirical Bayes(8) (EB) using the R package rstanarm (<https://cran.r-project.org/web/packages/rstanarm/index.html>). Empirical Bayes methods are procedures for statistical inference in which the prior probability distribution is estimated from the data. This approach stands in contrast to standard Bayesian methods, for which the prior distribution is fixed before any data are observed. For the estimation we used Markov Chain Monte Carlo (MCMC) sampling with a Gaussian prior for the regression coefficients. For the estimation two MCMC chains were used with 5,000 iteration each. The mean and the standard deviation of the Gaussian priors were obtained by estimating the same model by coordinate descent using a non-negativity constraint for the regression coefficients (i.e., the cell-type proportions) in the R package penalized(9).

### S1.3.2 Cell-type specific association studies with bulk data

For the cell-type specific association studies with bulk data we follow Shen-Orr et al.(10). Thus, the expression level in bulk are a weighted sum of the cell-type specific means with weights being equal to the cell-type proportions (e.g., if you have 40% neurons with mean expression of 0.1 and 60% glia with mean expression of 0.8, then the mean expression in bulk is 0.4*0.1 + 0.6*0.8). Thus,

$Y_{j}^{bulk}=\sum_{c=1}^{n_{c}} m_{j}^{c}P^{c}+E_{j}$ (2)

Where the *n_d_*×1 vector $Y_{j}^{bulk}$ are the bulk expression measurements for gene *j* for the *n_d_* donors, *c*=1..*n*_c_ indicate the cell types, *P*^c^ is the *n_d_*×1 vector with the proportion of cells of type *c* as estimated in step 1*,* $m_{j}^{c}$ is the mean expression level for gene *j* in cell-type *c*, and *E_j_* is the residual term for gene *j*. Note that this a is a standard multiple regression model with $m_{j}^{c}$being the regression coefficients and *P*^c^ the independent variables. Thus, the cell-type means can be estimated by a simple linear regression model without a constant. However, occasionally the model is written with a constant, whereby one of the cell-type proportions is omitted(11), which produces identical results(10, 12, 13).

The extension to test for differential expression between cases-and controls involves adding an interaction term:

$$Y_{j}^{bulk}=\sum_{c=1}^{n_{c}} m_{j}^{c}P^{c}+\sum_{c=1}^{n_{c}} m_{j(CC)}^{c}(CC\times P^{c})+E_{j}$$

If CC is coded 0 for controls the interaction term vanishes and the equation reduces to (2) meaning that $m_{j}^{c}$ now estimates the cell-type means in controls. If CC is coded 1 for cases, for cell-type *c* the mean in cases will differ $m_{j(CC)}^{c}\times P^{c}$ from the controls. Thus, the null-hypothesis for testing for differential expression of cell-type *c* is H_0_: $m_{j(CC)}^{c}=0$ for *c*=1..*n*_c_.

### S1.4 Demonstration bulk RNA-seq dataset

Bulk RNA-seq data was generated from the prefrontal cortex (Brodmann area 10) of 304 cases with a psychiatric disease and 291 controls. High quality RNA (RNA integrity number mean = 8.69, SD = 0.9590) was extracted from ~30mg of brain tissue using the AllPrep DNA/RNA extraction kit (Qiagen). The RNA-seq data was generated using the TruSeq Stranded Total RNA library kit with Illumina Ribo-Zero Plus with 0.7ug of total RNA from each sample as starting material. With the exception of that each reaction was scaled down, 0.7x the standard reaction volume was used, the vendors protocol was followed. In short, for each sample, ribosomal RNA was depleted from high integrity total RNA, cDNA was synthesized and indexed libraries were created. Up to 42 libraries were pooled in equal molarity and sequenced with paired end reads using a 2x150bp sequence configuration on a NovaSeq 6000 instrument (Illumina).

The sequenced reads were aligned with HISAT2 (v.2.1.0), files were processed by Samtools (14) and transcriptome assembly was performed with StringTie (v.1.3.3) (15) using the human reference genome GRCh37 from ENSEMBL. Following the initial transcriptome assembly with the reference genome, the StringTie merge option was used and the assembly was recreated using only the observed transcripts. In other words, all originally assembled transcriptomes, across all samples, were merged to create a project specific transcriptome including all transcript present in the investigated samples. Next, the abundance levels of all transcripts, for all samples, were re-quantified (stringtie -eB) to ensure that expression measures for specific transcripts are comparable across all samples.

All analyses included the covariates: sex and age, indicator variables to account for possible batch effects, and assay-related covariates such as total number of reads and the percentage of reads aligned. Furthermore, to account for remaining unmeasured sources of variation, 6 principal components that were obtained after regressing out the measured covariates from the bulk RNA-seq data, were included as covariates.

# S2 RESULTS

### S2.1 Alignment and quality control (QC)

We aligned reads with the “include-introns” option in cellranger that uses a gene transfer format (GTF) file that allows for intronic alignments. Table S1 provides sequencing statistics.

### Table S1. Sequencing statistics (separate file)

Table legend:

Variable: Variable label for sequencing statistic

Mean: Mean sequencing statistic

SD: standard deviations sequencing statistic

5% quant.: 5% quantile sequencing statistic

95% quant.: 95% quantile sequencing statistic

Variable description: Description sequencing statistic

Sample QC: Barcode rank plots plot the total barcode count (y-axis) against the rank of each barcode (x-axis) where the highest ranks have the largest totals. Barcodes for nuclei will have significantly more counts associated with them than the barcodes of background “noise”. A steep drop-off is therefore indicative of good separation between the cell-associated barcodes and noise-associated barcodes (e.g., Figure S1b). Conversely, a lack of steep drop-off may indicate low sample quality and many noise-associated barcodes (e.g., Figure S1b). To quantify, we used the fraction of reads associated with nuclei. Ten samples were omitted as less than 50% of the reads were associated with nuclei. This left 704,260 nuclei. For 8 of the 10 samples multiple libraries were available. For 2 of the 10 samples there was only one library so that the total number of controls decreased from 94 to 92 after this QC step.

### Figure S1 Examples of barcode rank plots


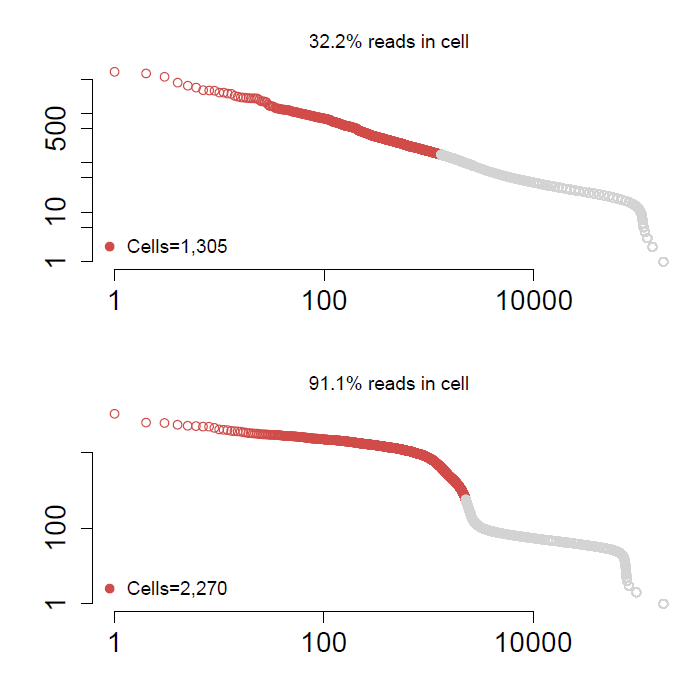


Nuclei QC: Two studies had many more nuclei than the others (309,079 and 182,645 versus the mean of 47,889 for the other 5 studies). To avoid that the clustering will be mainly be driven by these two studies we downsampled their nuclei by randomly selecting sequencing lanes (leaving 77,058 and 58,692 of the 309,079 and 182,645 nuclei). A total of 373,033 nuclei remained. After this QC step, the mean number of nuclei across the 7 studies was 55,005, SD=19,495, range 21,906-77,058).

Low-quality nuclei or empty droplets are likely to have few genes expressed and a small number of UMI counts, whereas nuclei multiplets are likely to have a high gene and UMI count as they capture expression levels of multiple nuclei. We removed 14,021 nuclei with fewer than 400 genes and more than 9,000 genes. These thresholds were chosen as they defined the extreme values of the distribution of number of genes per nucleus across the 7 studies(Figure S2, we used the log base 10 as this transformation was also used prior to performing cluster and association analyses). Next, we eliminated 3,447 nuclei with UMI counts < 500 and with UMI counts > 30,000 (Figure S3). Finally, we removed 2,419 nuclei with more than 5% of reads mapping to ribosomal genes as that may be an artifact stemming from sample preparation. This left 373,033-(14,021+3,447+2,419) = 353,146 nuclei for the cluster analyses.

### Figure S2. Distribution and QC threshold for number of genes per nucleus


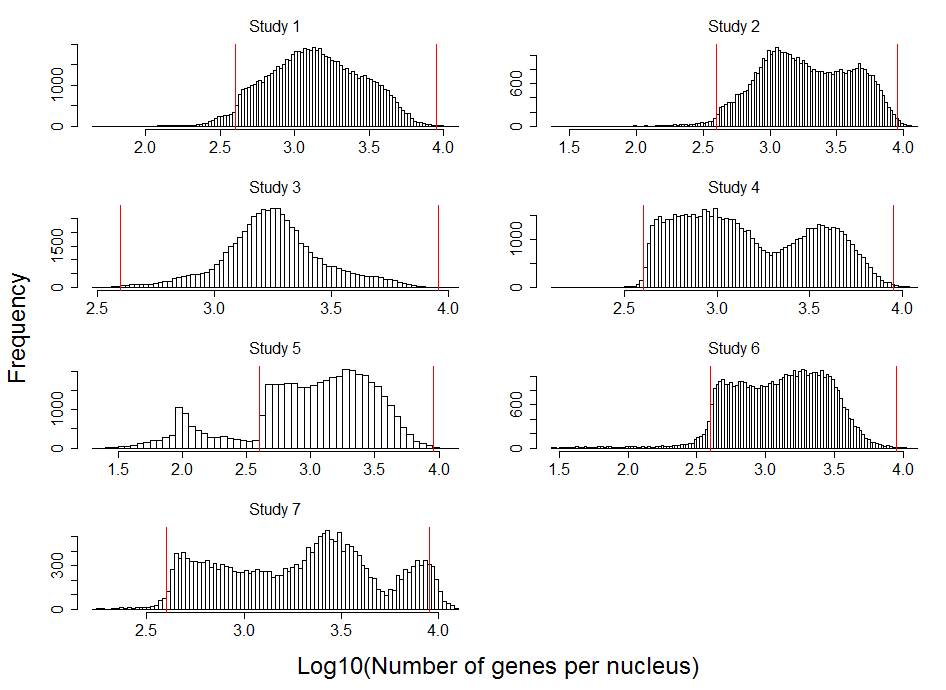


### Figure S3. Violin plot for number of genes per nucleus per cell-type


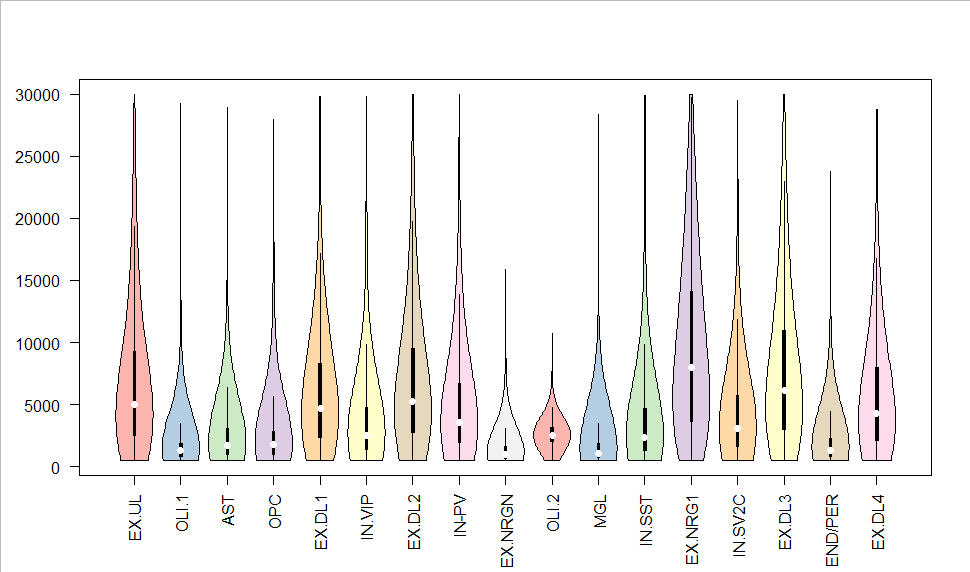


### Figure S4. Distribution and QC threshold for number of UMI counts per nucleus


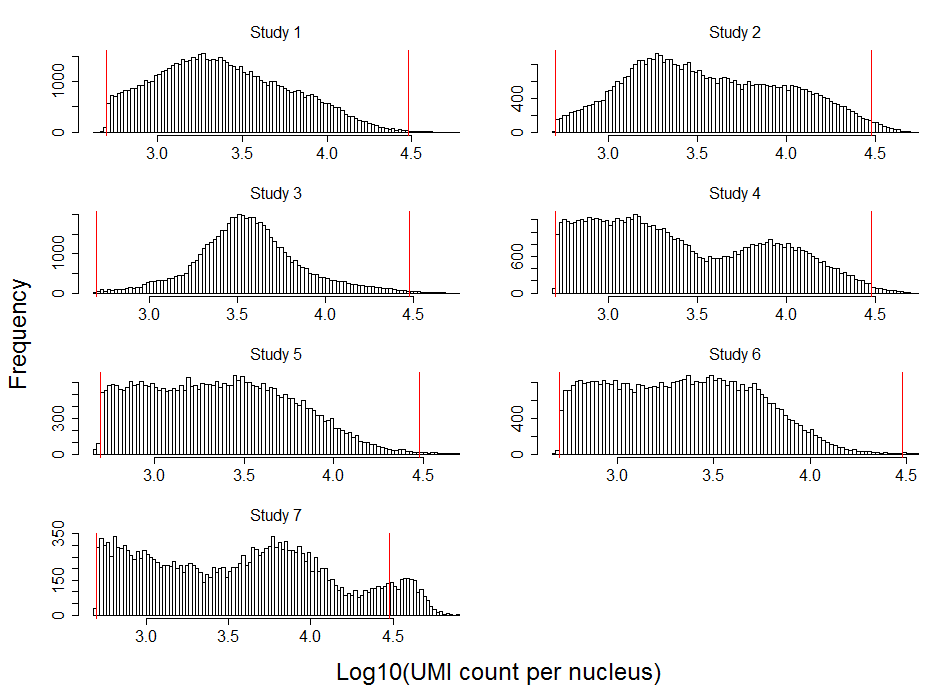


Gene QC: Our data comprised 36,601 genes. For the cluster analyses the 2,000 most highly variable genes were selected (see section S1.4).

Data transformations: UMI count data were log-normalized to obtain more normal distributions and reduce effects of possible outliers. Next, to give equal weight and avoid that highly-expressed genes dominate the cluster analyses, the data was scaled to have a mean expression across nuclei of zero and a variance of one.

### Figure S5. Violin plot for number of UMI counts per nucleus per cell-type


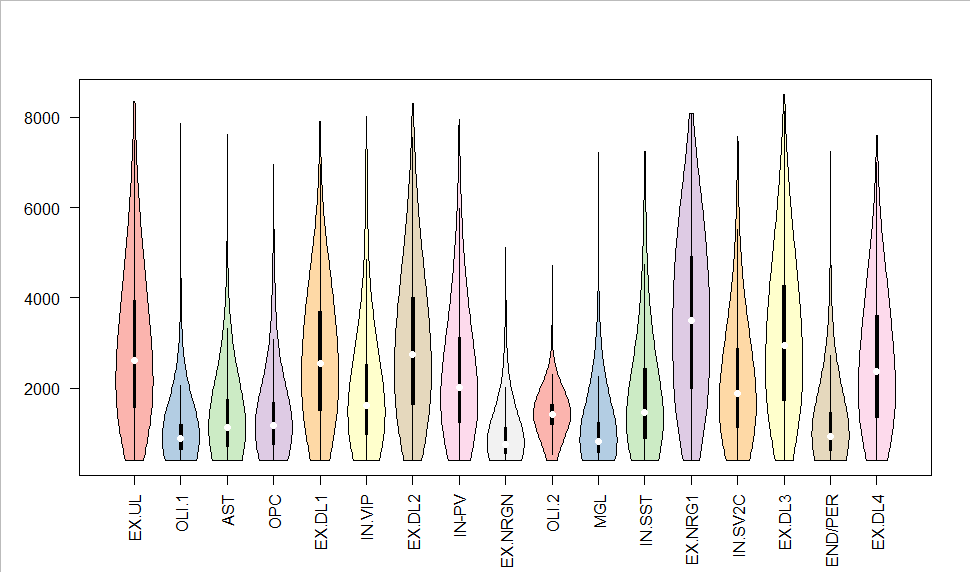


### S2.2 Cell-type identification and labeling

Figure S5 provides a dotplot for the markers used to label the cell-types where bigger dots correspond with higher expression levels. Figure S6a-e shows heatmaps of the overlap between the cell-type labels assigned in this study (Y-axis) and the label assigned in the five (X-axis) of the seven studies that provided nuclei labels. To determine the overlap we used all nuclei assigned to a cell-type cluster in our study and then calculated the proportion of those nuclei that were assigned to each of the cell-types in the original study. For example, if all nuclei labelled as astrocytes in our study would also be astrocytes in the original study the overlap would be one and if none of the nuclei labelled as astrocytes in our would be labelled astrocytes in the original study the overlap would be zero. Overlaps lower than one assigned 1% were left white. These results are further summarized in Table S2 provides for each of our cell-type clusters a list of the most highly expressed markers as well as the most frequently assigned original cell-type label in the five studies that provided labeled nuclei.

### Figure S6. Dotplot of gene expression markers used to annotate the clusters


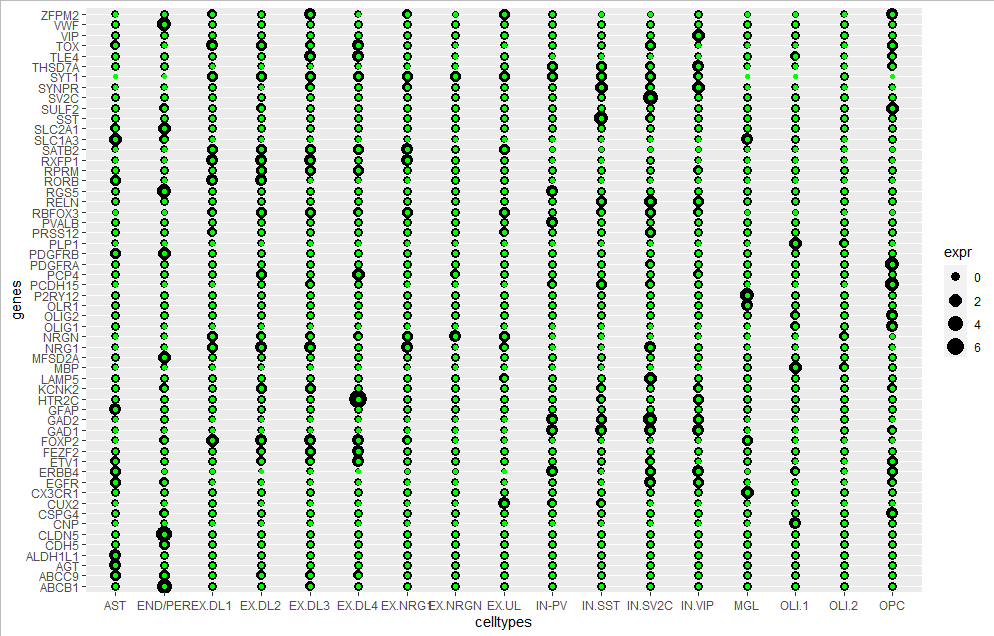


### Figure S7a-e. Heatmaps of the percent of overlap between cluster labels


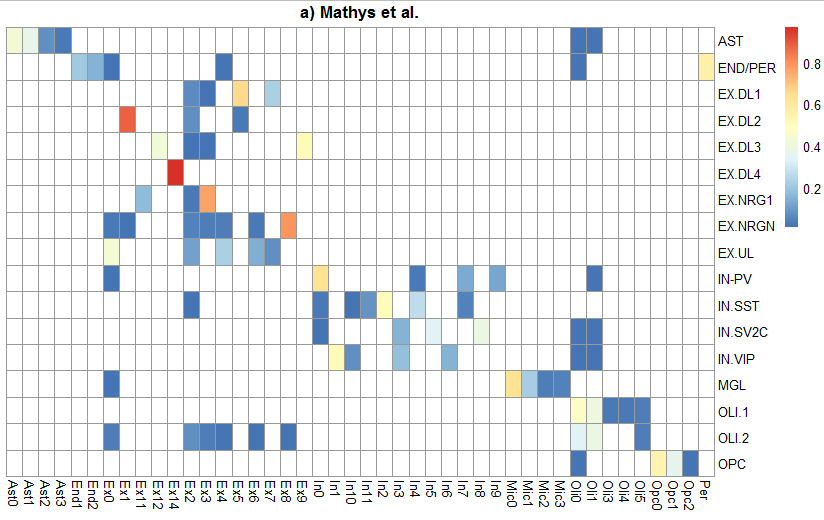


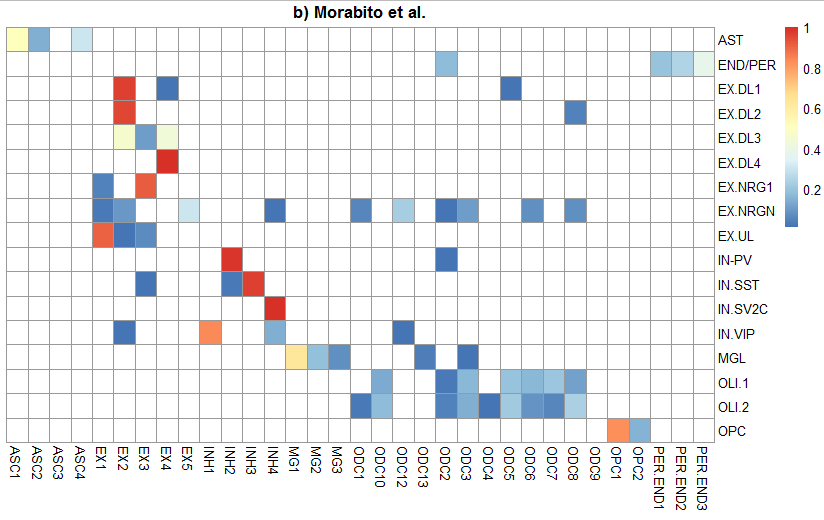


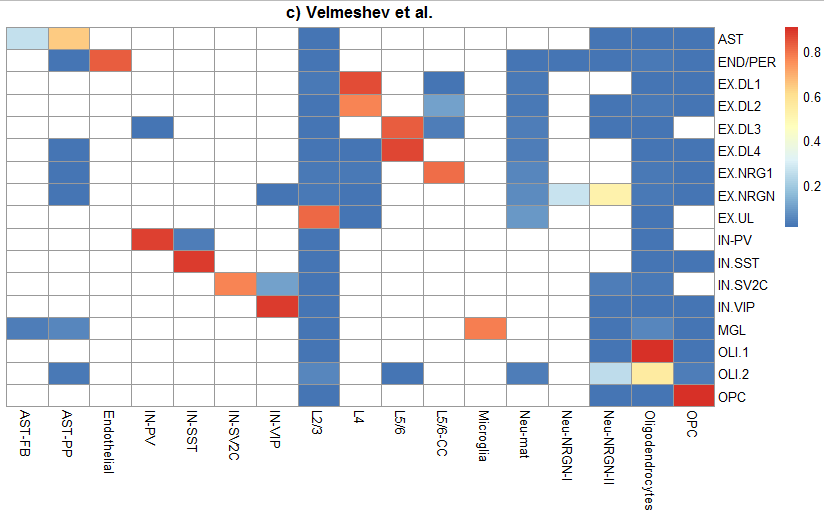


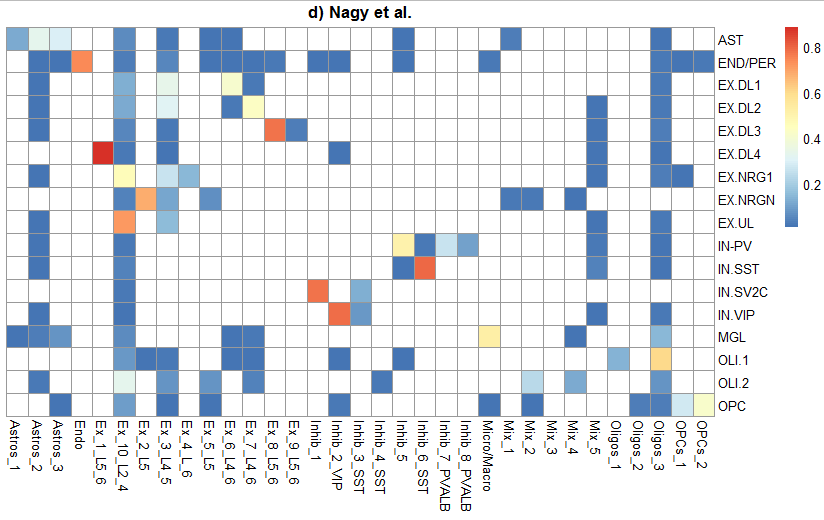


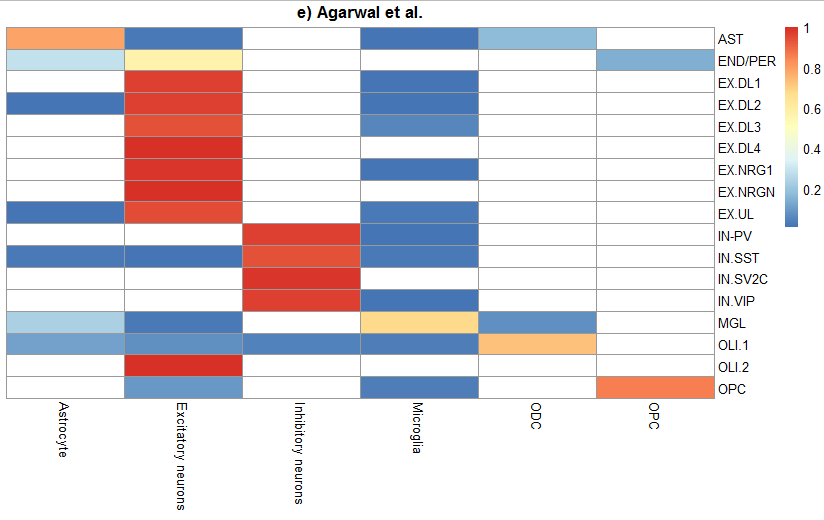


### Table S2. Cell-type labeling (separate file)

Table legend:

Label: Assigned cell-type label

Study 1 to study 5: Cell-type label assigned in the original study

Markers with expression levels > 1 SD above mean: Genes with expression levels > 1 SD above mean for listed cell-type

### Table S3. MAST identified cell-type panel markers (separate file)

Table legend:

Gene: Gene name

p_val : p_val (unadjusted)

avg_logFC : log fold-chage of the average expression between the two groups. Positive values indicate that the feature is more highly expressed in the first group.

pct.1 : The percentage of cells where the feature is detected in the first group

pct.2 : The percentage of cells where the feature is detected in the second group

p_val_adj : Adjusted p-value, based on bonferroni correction using all features in the dataset.

Cluster: The cluster (i.e., cell-type) for which the gene is a marker

### Table S4. Cerebral cortex reference panel (separate file)

Gene names are in row and cell-type label are in columns. Entries are standardized abundance levels

### Table S5. Grouping cell-type by principal components analysis

|  | PC1 | PC2 | PC3 | PC4 | PC5 | PC6 | PC7 | PC8 | PC9 | PC10 |
| --- | --- | --- | --- | --- | --- | --- | --- | --- | --- | --- |
| EX.UL | -0.54 | 0.041 | 0.045 | 0.118 | -0.07 | -0.13 | 0.083 | 0.023 | 0.054 | -0.01 |
| OLI.1 | 0.106 | 0.056 | 0.048 | -0.61 | -0.03 | -0.01 | -0.02 | 0.013 | 0.053 | 0.074 |
| AST | 0.463 | 0.206 | 0.235 | 0.452 | -0.14 | -0.01 | 0.171 | 0.119 | 0.215 | 0.298 |
| OPC | 0.057 | 0.031 | -0.79 | 0.051 | -0.24 | -0 | 0.019 | 0.002 | 0.029 | 0.046 |
| EX.DL1 | 0.057 | 0.066 | 0.032 | 0.056 | -0.01 | -0.11 | 0.105 | -0.01 | 0.01 | -0.74 |
| IN.VIP | -0.01 | 0.103 | 0.029 | 0.04 | -0 | 0.057 | 0.099 | -0.05 | -0.75 | 0.068 |
| EX.DL2 | -0.03 | -0.01 | 0.025 | 0.067 | -0.02 | 0.105 | -0.06 | 0.028 | 0.069 | -0.51 |
| IN.VP | 0.103 | -0.7 | 0.048 | 0.055 | -0.01 | -0.06 | 0.015 | 0.028 | 0.022 | -0.04 |
| EX.NRGN | 0.049 | 0.043 | 0.029 | 0.058 | -0.02 | -0.01 | -0.96 | 0.007 | 0.014 | 0.022 |
| OLI.2 | 0.076 | 0.039 | 0.043 | -0.62 | -0.03 | -0.01 | 0.077 | 0.028 | 0.052 | 0.069 |
| MGL | 0.056 | 0.022 | 0.086 | 0.033 | 0.861 | -0 | 0.012 | 0.003 | 0.018 | 0.02 |
| IN.SST | -0.01 | -0.66 | -0.01 | 0.023 | -0.01 | 0.056 | 0.016 | -0.02 | -0 | 0.082 |
| EX.NRG1 | -0.52 | 0.062 | 0.037 | 0.06 | -0.01 | 0.081 | -0.02 | 0.024 | 0.016 | 0.027 |
| IN.SV2C | 0.061 | -0.06 | 0.012 | 0.049 | -0.02 | -0.06 | -0.07 | 0.059 | -0.6 | 0.021 |
| EX.DL3 | -0.34 | -0.02 | 0.037 | 0.054 | 0.033 | 0.595 | 0.023 | -0.01 | 0.097 | 0.197 |
| END/PER | 0.047 | 0.012 | 0.018 | 0.038 | -0.01 | -0 | 0.006 | -0.99 | 0.03 | 0.027 |
| EX.DL4 | 0.26 | 0.013 | -0.02 | -0.03 | -0.03 | 0.766 | -0 | 0.01 | -0.06 | -0.2 |

Using an absolute loading of 0.5 as the cut-off, Table S5 suggest relatively high similarity in the expression of the (two) groups of OLIs, EX.DL3 and EX.DL4 excitatory neurons, and EX.DL1 and EX.DL2 excitatory neurons. Furthermore, IN.VIP and IN.SV2C interneurons, IN.SST and IN.PV interneurons, EX.UL and EX.NRG1 excitatory neurons.

## REFERENCES

1. van Dijk D, Sharma R, Nainys J, Yim K, Kathail P, Carr AJ, et al. Recovering Gene Interactions from Single-Cell Data Using Data Diffusion. Cell. 2018;174(3):716-29 e27.

2. Stuart T, Butler A, Hoffman P, Hafemeister C, Papalexi E, Mauck WM, 3rd, et al. Comprehensive Integration of Single-Cell Data. Cell. 2019;177(7):1888-902 e21.

3. Brennecke P, Anders S, Kim JK, Kolodziejczyk AA, Zhang X, Proserpio V, et al. Accounting for technical noise in single-cell RNA-seq experiments. Nat Methods. 2013;10(11):1093-5.

4. Finak G, McDavid A, Yajima M, Deng J, Gersuk V, Shalek AK, et al. MAST: a flexible statistical framework for assessing transcriptional changes and characterizing heterogeneity in single-cell RNA sequencing data. Genome Biol. 2015;16:278.

5. Leys C, Ley C, Klein O, Bernard P, Licata L. Detecting outliers: Do not use standard deviation around the mean, use absolute deviation around the median. Journal of Experimental Social Psychology. 2013;49(4):764-6.

6. Houseman EA, Accomando WP, Koestler DC, Christensen BC, Marsit CJ, Nelson HH, et al. DNA methylation arrays as surrogate measures of cell mixture distribution. BMC Bioinformatics. 2012;13:86.

7. Koestler DC, Christensen B, Karagas MR, Marsit CJ, Langevin SM, Kelsey KT, et al. Blood-based profiles of DNA methylation predict the underlying distribution of cell types: a validation analysis. Epigenetics. 2013;8(8):816-26.

8. B G, J G, I A, Brilleman S (2022). “rstanarm: Bayesian applied regression modeling via Stan.” R package version 2.21.3. rstanarm: Bayesian applied regression modeling via Stan. 2022.

9. Goeman JJ. L1 penalized estimation in the Cox proportional hazards model. Biom J. 2010;52(1):70-84.

10. Shen-Orr SS, Tibshirani R, Khatri P, Bodian DL, Staedtler F, Perry NM, et al. Cell type-specific gene expression differences in complex tissues. Nat Methods. 2010;7(4):287-9.

11. Montano CM, Irizarry RA, Kaufmann WE, Talbot K, Gur RE, Feinberg AP, et al. Measuring cell-type specific differential methylation in human brain tissue. Genome Biol. 2013;14(8):R94.

12. Chan RF, Turecki G, Shabalin AA, Guintivano J, Zhao M, Xie LY, et al. Cell Type-Specific Methylome-wide Association Studies Implicate Neurotrophin and Innate Immune Signaling in Major Depressive Disorder. Biol Psychiatry. 2020;87(5):431-42.

13. Zheng SC, Breeze CE, Beck S, Teschendorff AE. Identification of differentially methylated cell types in epigenome-wide association studies. Nat Methods. 2018;15(12):1059-66.

14. Danecek P, Bonfield JK, Liddle J, Marshall J, Ohan V, Pollard MO, et al. Twelve years of SAMtools and BCFtools. Gigascience. 2021;10(2).

15. Sahraeian SME, Mohiyuddin M, Sebra R, Tilgner H, Afshar PT, Au KF, et al. Gaining comprehensive biological insight into the transcriptome by performing a broad-spectrum RNA-seq analysis. Nat Commun. 2017;8(1):59.
